# BReastfeeding Attitude and Volume Optimization (BRAVO) trial: study protocol for a randomized controlled trial

**DOI:** 10.1186/s13063-016-1397-y

**Published:** 2016-06-02

**Authors:** Ary I. Savitri, Nikmah S. Idris, Wahyuni Indawati, Siti Rizny F. Saldi, Dwirani Amelia, Mohammad Baharuddin, Sudigdo Sastroasmoro, Diederick E. Grobbee, Cuno S. P. M. Uiterwaal

**Affiliations:** Julius Center for Health Sciences and Primary Care, Julius Global Health, University Medical Center Utrecht, Utrecht, The Netherlands; Department of Child Health/Center for Clinical Epidemiology and Evidence Based Medicine (CEEBM) Faculty of Medicine Universitas Indonesia/Cipto Mangunkusumo National General Hospital, Jl. Salemba 6, Jakarta, Pusat 10430 Indonesia; Budi Kemuliaan Health Institute, Jakarta, Indonesia

**Keywords:** Breastfeeding, Trial, Breastfeeding empowerment, Breastfeeding rate, Carotid artery wall intima-media thickness (CIMT), Arterial stiffness

## Abstract

**Background:**

A growing body of evidence shows the short-term benefits of breastfeeding, which include protection against infections, allergies, and lung diseases. However, evidence on the long-term benefits of breastfeeding is scarce and often conflicting. The BReastfeeding Attitude and Volume Optimization (BRAVO) trial is designed to study the effect of breastfeeding on early signs of later chronic diseases, particularly cardiovascular, respiratory, and metabolic risks later in life. In addition, the effectiveness of breastfeeding empowerment in promoting breastfeeding will also be evaluated.

**Methods/design:**

This study is an ongoing randomized trial in Jakarta, Indonesia, that began in July 2012. Pregnant women are being screened for their breastfeeding plan in the third trimester, and those with low intention to breastfeed are randomly allocated to either receiving an add-on breastfeeding-optimization program or usual care. Primary outcomes include breastfeeding rate, lung function, and blood pressure during the first year of life and vascular/cardiac characteristics, which will be measured at the age of 4 to 5 years. Child growth and infection/illness episodes are measured, whereas cognitive testing is planned for the children at 5 years of age.

**Discussion:**

To date, 784 women (80 %) have been randomized of the 1,000 planned, with satisfactory completeness of the 1-year follow up (90.1 %). Included mothers are of lower socioeconomic status and more often have blue-collar jobs, similar to what was observed in the pilot study.

**Trial registration:**

ClinicalTrials.gov, NCT01566812. Registered on 27 March 2012.

**Electronic supplementary material:**

The online version of this article (doi:10.1186/s13063-016-1397-y) contains supplementary material, which is available to authorized users.

## Background

The World Health Organization (WHO) aims to have newborns exclusively breastfed for 6 months [1]. However, it is estimated that globally only 38 % of infants are exclusively breastfed, and every year, 800,000 deaths among children under 5 years of age have been attributed to suboptimal breastfeeding [2]. These acute consequences are urgent, but concern has existed for decades that suboptimal breastfeeding may have lifelong health implications as well.

Breastfeeding is suggested to protect against infections, allergies, and lung disease, as well as overweight and obesity [3, 4], diabetes mellitus [5], cardiovascular risk factors [6, 7], and cardiovascular diseases [8, 9]. Such suggestions draw heavily on nonexperimental research in which, especially with breastfeeding, confounding bias is a major issue. Human breastfeeding experimentation in a fully explanatory way is impossible due to firm public and care preferences and strong adherence to WHO recommendations, and given a lack of equipoise for at least short-term benefits [10, 11]. However, numerous pragmatic randomized experiments had been done, primarily aimed to prolong breastfeeding duration, using a range of prenatal and/or postnatal interventions with different randomized designs and in a large variety of settings and populations [12–34]. By far, the largest and probably most informative is a cluster-randomized trial, the Promotion of Breastfeeding Intervention Trial (PROBIT), in over 17,000 women in Belarus, which showed that empowerment raised breastfeeding rates and decreased infant gastrointestinal infection and atopic eczema rates, thus clearly underpinning short-term benefits [35]. However, later nonrandomized analyses on that trial did not show protection against obesity and high blood pressure [36–38], allergies and asthma [39], and behavior problems [40].

Indonesia is a low-income to middle-income country with a population of 250 million and 4 to 4.5 million births per year. Indonesia lags behind WHO recommendations with only 15 to 40 % exclusively breastfeeding [41–43]. Indonesia is experiencing an epidemiological transition, and it is among the regions identified to face a strong rise in noncommunicable diseases, including atherosclerotic disease and a significant prevalence of chronic obstructive pulmonary disease [44, 45]. Much potential exists for an improvement of breastfeeding practices, as breastfeeding empowerment programs have been shown effective in various Southeast (SE) Asian countries, although not in Indonesia [46–48]. Modifiable predictors of nonexclusive breastfeeding in SE Asian countries have been identified and include the absence of prenatal breastfeeding planning, working by mothers, and the noninvolvement of fathers in the breastfeeding planning [49]. Generally, breastfeeding-promotion programs are probably more successful in achieving exclusive breastfeeding in lower income countries like Indonesia than in economically more affluent societies [50]. Nevertheless, due to the specific local mix of cultures, religions, ethnic backgrounds, and limited resources, the effectiveness of empowerment programs that worked elsewhere, even in SE Asia, cannot be taken for granted in Indonesia.

A new randomized breastfeeding trial is currently being conducted in Jakarta, Indonesia. The Breastfeeding Attitude and Volume Optimization (BRAVO) trial (clinicaltrials.gov NCT01566812) aims to individually randomize 1000 women in later pregnancy, when decision making about breastfeeding is imminent. In a pilot study, a stepped-screening procedure for eligibility was tested in 207 pregnant women, of whom 56 % intended to breastfeed less than 2 months, and 38 % were willing to be randomized [51]. Like virtually all reported breastfeeding empowerment trials, BRAVO questions whether breastfeeding can be optimized toward WHO recommendations. However, in BRAVO, the principal aim is to study breastfeeding effects on early life signs of later life cardiovascular and cardiometabolic risks, and respiratory risks that have become particularly pressing in Indonesia [42, 52]. This aim has led us to choose individual rather than the commonly used cluster randomization for intensive individual prenatal and postnatal empowerment intervention for maximal breastfeeding contrasts, and to choose a trial size and measurements specifically aimed at the detection of cardiovascular, cardiorespiratory, and cardiometabolic effects in early childhood. BRAVO aims to measure the effects of breastfeeding on the developing cardiovascular organ system itself.

To date, BRAVO has included and randomized 780 pregnant women in their third trimester. The BRAVO design is herein presented, along with a cross-sectional description of both the screened and enrolled populations.

## Methods/design

### Study design and setting

**Fig. 1:**
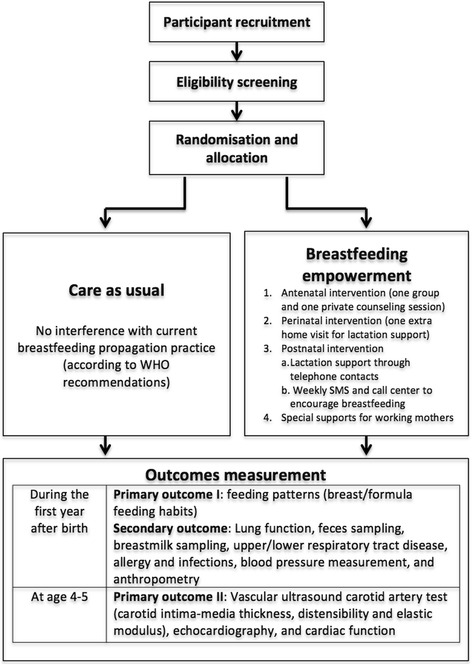
Flowchart of the study process. SMS, short message service; IGF-1, insulin-like growth factor 1; IGFBP-3, insulin-like growth factor binding globulin 3

BRAVO is a pragmatic, parallel, group-randomized trial that began in July 2012. The report of this study protocol was made conforming to the SPIRIT 2013 Statement (Standard Protocol Items: Recommendations for interventional trials) [53] (see Additional file 1). The study process is illustrated in Fig. 1.

During pregnancy, eligible women are randomly allocated either to receive a program aimed at add-on breastfeeding optimization during pregnancy and the first 6 months after delivery or to receive primary care as usual. After randomization, a first cardiovascular assessment will be made when the children are between 4 and 5 years of age.

Women under primary or referred care are screened for eligibility. Women are recruited from Budi Kemuliaan Hospital, a private specialized referral center for maternal and child care in the municipality of Jakarta (Director MB), which has a delivery rate of 7,000–8,000 newborns/year. It performs service delivery, training (midwives), education, and research. The hospital has ten staff members with Good Clinical Practice certificates. The attending women are partly mobile, but many live in the vicinity and have the hospital as first choice. Budi Kemuliaan features a home-visit program by midwives in training. Restriction to women with Budi Kemuliaan as first choice ensures some 3,000/year for inclusion. Women are also recruited from local primary care centers, including primary care centers in the Senen and Jatinegara districts. This study was ethically approved by the Institutional Review Board of the Faculty of Medicine University of Indonesia/Cipto Mangunkusumo General Hospital (reference number: 913/UN2.F1/ETIK/X/2015).

### Stepped eligibility screening, recruitment, and informed consent

BRAVO has a stepped-screening procedure for eligibility, of which the details, including the inclusion criteria, are given in Table 1. Criteria for eligibility include pregnant women under primary or referred healthcare who intend to breastfeed their newborn shortly or not at all.

**Table 1:** BRAVO eligibility screening steps during gestation

Step	Purpose	
1	Plans breastfeeding	Gestation week 28–36: midwives provide a short questionnaire to measure plans for breastfeeding duration ≤ 2 months 2 to ≤ 4 months 4 to ≤ 6 months > 6 months Women indicating ≤ 2 months are eligible
2	Stability residence	From the group of women that were selected under Step 1, a further selection is made of those living in the vicinity of the hospital
3	Inclusion criteria	1. Residing in vicinity of participating hospital or primary care centers 2. Telephone communication possible with mother, concerning present pregnancy 3. Uncomplicated (no morbidity requiring hospitalization or intensive care, e.g., eclampsia) 4. No major fetal congenital disease 5. No known HIV in mother
4	Passed steps 1–3	Formal invitation of women to participate in BRAVO

Women who, after randomization, have babies that are stillbirth, of premature birth (birth weight < 2500 g, gestational age < 37 weeks), or have congenital heart disease or multiple congenital anomaly are not excluded and will be part of the intention-to-treat analysis

Midwives or primary care workers in charge of routine pregnancy care do the recruitment during visits. Informed consent will be obtained from all participants. Eligible women are first informed about BRAVO and, then, are formally invited to participate. Women are given 1 week to decide and are asked to inform their midwives or primary care physicians about their decision at the next visit or by telephone calls if they have then not decided yet. Women who decide to participate are asked to sign a BRAVO informed consent form (see Additional file 2).

### Pilot study

Prior to BRAVO, a pilot study was conducted to assess the feasibility of the procedures related to the screening, invitation, informed consent, and randomization (see Background). The pilot focused on the active roles of the midwives and primary care physicians, both from a practical and theoretical viewpoint. Women were subjected to procedures for antenatal and postnatal breastfeeding support as planned (see Interventions). Experiences of women and caretakers were registered and used for fine-tuning of the empowerment intervention. As caretakers are responsible for both BRAVO conduct and future empowerment implementation, their needs and comments were maximally accommodated.

### Interventions

1. **Care as usual**
  Care as usual means that there is no interference with breastfeeding propagation practice in current Indonesian care, which, in general, follows the WHO recommendations for successful breastfeeding. The implementation of these recommendations varies across hospitals and primary care centers according to resource availability. Generally, education about breastfeeding is given individually by attending midwives or physicians during antenatal visits.
2. **Breastfeeding empowerment**
  Randomized trials in Southeast Asia have shown that empowerment effectively raises exclusive breastfeeding rates [47]. For BRAVO, these programs were adapted in both language and culture to the Indonesian setting. Clearly, optimal empowerment comes from both antenatal and postnatal intervention [47]. The BRAVO program consists of antenatal, perinatal, and postnatal intervention, and special supports for working mothers, including the provision of breast pump equipment when necessary and active support at workplaces. A lactation manager coordinates breast pump provision and gives written advocacy to employers to provide a lactation room and working hours that allow mothers to express their breast milk.
  During late pregnancy, women in the intervention group are subjected to one private and one group-counseling session. To avoid intervention contamination, the antenatal visit in the empowerment intervention is handled by a particular group of midwives or primary care physicians who underwent a standardized breastfeeding-counseling training during BRAVO preparation, while the care-as-usual group members have routine antenatal visits to regular caretakers. At private counseling, conducted within 28–37 weeks of gestational age, women and families are enabled to visit a lactation counselor for 30 minutes, mainly to build motivation for breastfeeding. A set of leaflets addressed specifically to mothers, husbands, or grandmothers is given at the end of the session. At group counseling, women are subjected to a commercially available video (http://injoyvideos.com/) based on a 16-minute educational video entitled “14 steps to better breastfeeding” (Injoy Videos, Boulder, CO). The video is voiced over in Indonesian language and demonstrates correct positioning, latch on, and breast care. Closest family members (husband and/or women’s mother) are also invited to watch the video. Group counseling also allows mothers to share opinions with peers and mutual encouragement.
  Empowerment intervention continues around delivery. Apart from early initiation and lactation support as part of routine care in Budi Kemuliaan Hospital and primary care centers, the intervention arm receives one extra home visit from appointed midwives/breastfeeding counselors to establish breastfeeding, address problems, and check preparations at home.
  As part of the postnatal intervention, women in the intervention group are visited at home by caretakers within the first postnatal week, both with usual care and experimental lactation support. A second lactation support visit takes place within week 1–2 after delivery. Thereafter, midwives and primary care workers have telephone contact once in 2 weeks in the first month and once a month thereafter for lactation support until 6 months after delivery. A centrally managed, regular, short message service (SMS) to encourage breastfeeding practice is sent weekly. A breastfeeding hotline or call center managed by midwives is set up to anticipate urgent questions or problems of the mothers. Mothers in the intervention group who return to work before 6 months after delivery receive additional counseling on preparing, storing, and feeding their babies with expressed breast milk. Also, letters are written to formally advocate for the mothers’ employer to facilitate breastfeeding continuation by providing secluded space, working hours, and environments that allow for expressing breast milk. A breast-milk pump rental is arranged on request.

### Trial retention

To promote trial retention, all mothers, irrespective of the intervention arm, receive reimbursement for their travel expenses to the primary health care or research hospital, and infants who are not covered by government health insurance obtain free basic immunization. Investigators also set up a stepwise standard procedure for mothers who do not turn up for the routine health check; this procedure includes at least two telephone reminders and home visits if mothers were not able to come for the outcome measurement.

### Baseline trial measurements

Table 2 shows the baseline parental and child characteristics that are measured in BRAVO.

**Table 2:** Baseline characteristics of the parents and of the child’s birth

Baseline parental characteristics	Unit	Source
Postal code and contact address		Questionnaire
Age	Years	Questionnaire
Marital status	Give response classes?	Questionnaire
Pre-pregnancy weight	kg	Questionnaire
Number of (and age of) other children	*N*, years	Questionnaire
Substance use (smoking, alcohol, etc.)	Yes/no	Questionnaire
	Number/day?	
Socioeconomic status (highest education of father/mother)	Give response classes?	Questionnaire
Occupational status (mother/father)	Give response classes?	Questionnaire
Nutrition during gestation		Questionnaire
Family history of chronic disease ((chronic) lung disease, diabetes mellitus, cardiovascular disease)		Questionnaire
Maternal and paternal disease history. ((chronic) lung disease, diabetes mellitus, cardiovascular disease)		Questionnaire
Children’s birth characteristics		
Gestational age in weeks	Weeks	Midwife registry
Birth weight in grams	Grams	Midwife registry
Birth length in centimeters	Centimeters	Midwife registry
1-minute and 5-minute Apgar scores	Values	Midwife registry
Specifics of gestation: (pre-)eclampsia, diabetes mellitus		Midwife registry
Specifics of delivery: caesarian section, breech position, and vacuum/forceps extraction		Midwife registry
Specifics of neonatal morbidity: Neonatal Intensive Care Unit (NICU)/special nursery care requirements, including sepsis, respiratory distress, and hyperbilirubinemia		Midwife registry

### Endpoints

BRAVO deals with studying every child’s first year; in addition, it will follow children until at least their 5^th^ year of life for carotid artery wall measurements, pulmonary function, anthropometry, and disease history. A general scheme of measurements and timing throughout BRAVO follow-up is presented in Table 3. Outcome assessors and data analysts are being blinded about the participants’ assignment to interventions. Retention of mothers and children at the age of 5 years will be optimized by proactively contacting the mothers by telephone or in writing.

**Table 3:** Schedule of enrollment, interventions, and assessments

	Study period															
	Enrollment	Allocation	Post-allocation													Close-out
TIME POINT	Gestation week		Birth	month												
	*24–36*	32–36	*1*	*2*	*3*	*4*	*5*	*6*	*7*	*8*	*9*	*10*	*11*	*12*	*…*	*48–60*
ENROLLMENT:																
Eligibility screen	X															
Informed consent	X															
Allocation		X														
INTERVENTIONS:																
Antenatal intervention		X														
Postnatal home visit			X													
Biweekly postnatal contacts																
Postnatal visits			X	X		X		X			X			X		
Telephone calls			X	X	X	X	X	X	X	X	X	X	X	X		
*SMS*			X	X	X	X	X	X								
ASSESSMENTS:																
Maternal ad family data		X														
Lung function SOT			X					X								
Lung spirometry														X		X
Feces sampling			X	X		X		X			X			X		
Breastmilk sampling			X	X		X		X			X			X		
Diary upper/lower RTD, allergy, infections			X	X	X	X	X	X	X	X	X	X	X	X	X	
Blood pressure measurement			X	X		X		X			X			X		X
Anthropometry			X	X		X		X			X			X		X
Vascular US carotid artery test																X
Pulse-wave velocity/stiffness																X
Echocardiography cardiac function																X
IGF-1, IGFBP-3, leptin levels, fibrinogen*														X		X

*SMS* short message service, *SOT* single occlusion technique, *RTD* respiratory tract disease, *US* ultrasound, *IGF-1* insulin-like growth factor 1, *IGFBP-3* insulin-like growth factor binding globulin 3

Primary outcome I includes the monthly questionnaire/telephone call to discuss feeding patterns (breast/formula feeding habits).

Primary outcome II includes the vascular wall characteristics at 4 years old (thickness of carotid intima-media, distensibility, and elastic modulus) (see Table 4).

**Table 4:** Cardiovascular endpoint measurements

	Methods	Arterial segment	Timing
Vascular ultrasound	Carotid artery		48 to 60 months
Arterial stiffness: pulse wave velocity, augmentation index	Arteriography		48 to 60 months
Blood pressure	Oscillometric device	Brachial artery	2, 4, 6, 9, and 12 months, 48 to 60 months
Cardiac function: cardiac output, LV systolic/diastolic function, RV diastolic function	Echocardiography	-	48 to 60 months

*LV* left ventricle, *RV* right ventricle

The secondary outcomes include the blood pressure, lung function/respiratory tract disease, infection, allergy, obesity, growth and development, cardiac function, breast-milk analysis (especially the fat content), and the gut/airway microbiome.

For subjects who discontinue or deviate from the study protocol but still live in the Jakarta area, home visits are conducted to collect data for breastfeeding habits, history of illness in the past 1 month, and growth data taken from the infant health card.

### Data management, randomization, statistical power, and data analysis

All trial data are gathered through web-based data entry sheets (www.bravo-trial.com) by research nurses/assistants. Data management is the responsibility of the Department of Child Health/Center for Clinical Epidemiology and Evidence-Based Medicine (CEEBM) Faculty of Medicine University of Indonesia/Cipto Mangunkusumo National General Hospital in Jakarta, Indonesia. All investigators who belong to the BRAVO study group have access to the final trial database through the data manager (SRFS). Local principal investigators (NSI, WI) are also granted direct access to the trial database for monitoring. Public availability of clinical trial data is a pending debate [54] and until decided, any BRAVO data usage is decided by consensus of the BRAVO study steering committee (NSI, CSPMU, SS, WI, and DEG). Randomization is centrally performed using computer-based random lists for simple parallel groups. All participating women are given individual study numbers, and their personal information is collected, shared, and maintained in order to protect confidentiality before, during, and after the trial.

BRAVO is restricted to pregnant women who, from their own free will, plan to give breastfeeding for no longer than 2 months, if at all. Forty percent of all pregnant women were estimated to meet that criterion. Thus, for every four eligible women, 10 pregnant women have to be screened, assuming that the number of women lost to exclusion is negligible. The effect of the empowerment program on the increase of exclusive breastfeeding was the first required estimate. From a Southeast Asian trial that used no restrictions on women’s plans to breastfeed for short times, exclusive breastfeeding proportions in the experimental group were expected to be at least 1.5 times higher than in the care-as-usual group. This relative risk was reported throughout the first 6 neonatal months but decreased from 83 % to 53 % at 14 days postpartum and ultimately to 20 % in the experimental group, and to 0 % in the usual-care group at 6 months. In 5-year-old healthy children, breastfeeding versus formula feeding was suggested to have an effect on carotid artery wall intima-media thickness of 20 μm, at an average CIMT of 386 μm (SD 37). Translating to BRAVO, a comparison of 54 women in an exclusive breastfeeding group and 54 women in an exclusive bottle-feeding group would allow detection of a 20-μm group difference in carotid IMT, with 80 % power and alpha = 0.05. Obviously, BRAVO does not yield randomized groups of exclusive breastfeeding and exclusive bottle-feeding groups. Due to contemporary uncertainties about the effects of empowerment on breastfeeding and ultimately on the cardiovascular system, it was assumed that a group that was at least ten times bigger was to be randomized. BRAVO, therefore, aimed to randomize 1000 eligible pregnant women, requiring screening of approximately 2500 pregnant women. A trial of 1000 women can detect small differences in breastfeeding practices between intervention groups. An estimated 500 women randomly allocated to each group would allow for the statistical detection of a nominal difference of almost 10 % in exclusive breastfeeding proportions, with 90 % power and a statistical significance level of 0.05 (PASS 2008). From a public health point of view, a difference of 10 % or higher was considered meaningful.

With respect to data analysis, both descriptive and baseline prognostically important characteristics will be tabulated against intervention strategy. The chance of successful exclusive breastfeeding for 6 months (yes/no) will be compared between the empowerment and care as usual group by calculating the relative risk with 95 % confidence intervals. As an absolute risk estimate to support public health policy making, the number needed to treat (by breastfeeding empowerment) and 95 % confidence intervals will be calculated. The duration of breastfeeding will be studied as a function of the interventions by using linear regression with (transformed) duration as the dependent variable and a group indicator as the independent variable. Should there be post-randomization differences that would hamper proper interpretation, regression modeling will be used to adjust all findings for baseline prognosis (propensity score or inverse probability weighting techniques).

The effects of intervention on cardiovascular measurements will be studied using linear regression techniques with cardiovascular measurements as the dependent variables and a group indicator variable as the independent variable. All regression models will also be used for adjusting for baseline noncomparability when necessary. All analyses will be performed according to the intention-to-treat principle, such that all randomized women will be analyzed by their randomized allocation. Possibly, biased missingness of the 5-year outcome data will be assessed using additional tables of baseline data comparing the available and missing data, and the above-described analytical techniques will be applied for dealing with prognostic group differences [55]. Missing data will be analytically addressed using multiple imputation regression techniques.

### Results of screening, enrollment, and preliminary follow-up

Table 5 shows the characteristics of women who were screened for BRAVO. The ages of the included and excluded women were similar. Included women were more often of low socioeconomic status. Although there was no statistically significant difference in the proportion of working mothers between the excluded and included women, included women more often had blue-collar employment. So far, the 1-year follow-up is 90.1 % complete.

**Table 5:** Characteristics of screened women (*n* = 1234)

	Excluded *N* = 707	Included *N* = 527	*P* value
Age	31.3 (5.7)	30.3 (5.9)	0.30^1^
Socioeconomic status			<0.001^2^
Low	78 (12.7)	117 (22.9)	
Middle	432 (70.5)	358 (70.1)	
High	103 (16.8)	36 (7)	
Number of pregnancy			0.70^2^
1	233 (37.8)	212 (41.2)	
2	218 (35.3)	173 (33.7)	
3	112 (18.2)	87 (16.9)	
>3	54 (8.8)	42 (8.2)	
Working mother	213 (34.6)	152 (29.7)	0.08^2^
Blue-collar job	49 (23.9)	55 (37.7)	0.006^2^
Previous breastfeeding	310 (43.8)	248 (47.1)	0.14^2^

Values are means with standard deviations for continuous variables and percentages for frequencies. In case of skewed data (*), the median with the interquartile range was presented

^1^Independent samples *t*-test, ^2^Chi-Square test

## Discussion

To our awareness, BRAVO is the first randomized breastfeeding empowerment study that is specifically focused on estimating the early life cardiovascular and cardiometabolic consequences of breastfeeding. Currently, at almost 80 % of the intended enrollment, the first part of BRAVO has been shown feasible and highly successful.

A strong feature of BRAVO is the number of individually randomized pregnant women; to our knowledge, this trial is one of the larger of its kind in a low-resource setting. The principal aim of BRAVO, which is to study the effects of breastfeeding on the early life development of cardiovascular function and structure, sets it apart from other trials that were primarily designed to measure the effects of empowerment on breastfeeding proportions. A major challenge in trials is to achieve accurate and complete follow-up. This is particularly challenging in BRAVO, as it deals with young families in a highly mobile culture. In BRAVO, the follow-up, to be done for the first year of life, is designed to follow regular care (vaccination scheme), is proactive, and includes home visits. So far, follow-up completeness is satisfactory, although the final results will have to be awaited. BRAVO, like all other breastfeeding trials, is pragmatic, so its ultimate success depends on the contrast in breastfeeding between intervention arms that can be achieved. This contrast will still be a function of distributions of breastfeeding duration in both arms and, importantly, on the distributions of bottle and other infant feeding in both arms. Obviously, BRAVO registers breastfeeding duration in both arms as well as the introduction of other feeding habits. The fact that BRAVO, as with other trials, can only study cardiovascular effects as a function of the overall breastfeeding contrast is a limitation that cannot be circumvented by design. On the other hand, the pragmatic character of BRAVO will allow for easier implementation of beneficial results, if found. In principle, a threat to the BRAVO design is that women who are randomly allocated to care as usual will, knowing about the study objectives, change their breastfeeding plans, thus leaving a smaller contrast between the experimental intervention arms. An alternative would have been pre-randomization (first randomize, then obtain informed consent), such as done in Zelen’s design [56]. However, since BRAVO focuses on women who during pregnancy have already planned to provide for a maximum period of 2 months breastfeeding, pre-randomization, with its associated medical ethical implications, was not considered necessary. In BRAVO, individual women are randomized, rather than clusters of women within, for instance, primary care practices. Monitoring of breastfeeding motivation takes place on an individual basis and is performed by the study nurses. The effects of close monitoring are not expected to go easily for all pregnant woman. Although, in principle, a risk of intervention contamination exists, BRAVO uses individual rather than cluster randomization for a couple of reasons. First, the full intervention, both prenatal, perinatal, and postnatal, is well outside the realm of usual care, as it requires high levels of organization (group counseling, home visits). Although cluster randomization was considered, only limited numbers of clusters were feasible in the BRAVO setting, which would have substantially increased the chance of baseline prognostic noncomparability, a much bigger threat to the overall purposes than contamination. A cluster-randomized design would have required larger numbers of pregnant women, in whose offspring highly specialized cardiovascular and respiratory measurements were considered unfeasible. Finally, a cluster-randomized design bears intrinsic prior uncertainties, such as ultimately achieved (differences in) cluster sizes and unknown within-cluster correlations. Although such variation can be dealt with in analysis, the BRAVO design with individual randomization was considered a priori a more robust approach, specifically concerning its overall aim, the study of cardiovascular and cardiometabolic consequences of breastfeeding.

Obviously, breastfeeding randomization with its claimed beneficial health outcomes can only be considered within certain ethical boundaries. In principle, BRAVO does not interfere with current practice and personal maternal choices regarding children’s nutrition; instead, it applies a breastfeeding intervention that is meant to improve breastfeeding practices. As alluded to above, many examples exist of such randomized breastfeeding empowerment studies, including in (Southeast) Asian countries [48]. The ethical justification of such trials has been uncertainty or equipoise with regard to the effectiveness of empowerment programs on breastfeeding rates. As in the Indonesian setting, where the effectiveness is currently unknown, it is uncertain whether Indonesian women allocated to care as usual are withheld better care, and whether an alternative of empowerment is better is simply unknown. Therefore, as there is equipoise, randomization is considered justified. Moreover, women in BRAVO are selected such that they, by their own choice, plan to provide breastfeeding for a maximum of 2 months, if at all. If BRAVO were not conducted, all pregnant women would receive no empowerment other than that currently available under care as usual.

The first results of BRAVO show that some 40 % of screened women participated in BRAVO, which is slightly above the expected number. The enrolled and randomized women were more often of low socioeconomic status and more often had blue-collar employment, since they were more likely to have no plans for extended breastfeeding. This was in line with findings from our BRAVO pilot study [51] and previous studies, which showed an inverse relationship between breastfeeding rate and the socioeconomic status [57–59]. The decline rate of participants did not differ according to the women’s socioeconomic status.

We believe that BRAVO is important for a number of reasons. First, since it is performed in Indonesia and strongly embedded in Indonesian maternal and child care, its results with regard to possibilities for breastfeeding optimization will be readily implementable locally. Second, suggestions about the beneficial effects of breastfeeding on cardiovascular and cardiometabolic risks later in life require stronger supportive evidence [60]. We believe that the BRAVO design presented herein can provide such evidence.

We conclude that a complex randomized trial like BRAVO can be successfully conducted in a low-resource setting like Indonesia, where solutions for low exclusive breastfeeding proportions and evidence about both short and long-term benefits are urgently needed.

### Trial status

The study is currently recruiting participants. By 19 April 2016, of the 1000 participants required, 853 women had been randomized. The estimated study completion, including the measurement of cardiovascular outcomes, is July 2021.

## Abbreviations

BRAVO, Breastfeeding Attitude and Volume Optimization; CEEBM, Clinical Epidemiology and Evidence-Based Medicine; CIMT, carotid artery wall intima-media thickness; PROBIT, Promotion of Breastfeeding Intervention Trial; SE, southeast; SMS, short message service; WHO, World Health Organization.

## Additional files

Additional file 1: SPIRIT 2013 checklist: Recommended items to address in a clinical trial protocol and related documents. We have indicated the page number where each item is addressed. (DOC 120 kb) Additional file 2: Informed consent form. This informed consent form contains details about the study that are used to inform the women. Women who agree to participate in the study are asked to sign this form. (DOC 49 kb)
