# Supplementary material for: BReastfeeding Attitude and Volume Optimization (BRAVO) trial: study protocol for a randomized controlled trial
Source: Trials. 2016 Jun 2;17:271. doi: 10.1186/s13063-016-1397-y (PMC4890480; doi:10.1186/s13063-016-1397-y)
Supplement: Additional file 2: — Informed consent form. This informed consent form contains details about the study that are used to inform the women. Women who agree to participate in the study are asked to sign this form. (DOC 49 kb) [file 13063_2016_1397_MOESM2_ESM.doc]

**Lembar Persetujuan (*Informed Consent)***

**Keikutsertaan Ibu/Anak dalam Penelitian:**

***BReast feeding Attitude and Volume Optimization (BRAVO) trial:***

***A randomized breast feeding optimization experiment***

**Peneliti Utama:** Dr. Nikmah Salamia Idris, SpA^1,2^, Dr. Wahyuni Indawati, SpA^2^, Siti Rizny Fitriana Saldi, Apt., MSc. Epid^1^, Prof. DR. Dr. Sudigdo Sastroasmoro, SpA (K)^1,2^, Prof. Cuno S.P.M. Uiterwaal, MD^3^, Dr. M. Baharuddin, SpOG, MARS^4^

1. ***Center for Clinical Epidemiology and Evidence-Based Medicine*** (CEEBM) Fakultas Kedokteran Universitas Indonesia – Rumah Sakit Cipto Mangunkusumo, Jakarta; &
2. **Departemen Ilmu Kesehatan Anak,** Fakultas Kedokteran Universitas Indonesia – Rumah Sakit Cipto Mangunkusumo, Jakarta; &
3. ***The Julius Center for Health Science and Primary Care*,** University Medical Center Utercht, Belanda; &
4. **Rumah Sakit Budi Kemuliaan**, Jakarta, Indonesia

**Lembar persetujuan ini terdiri dari dua bagian:**

- Lembar Informasi (berisi informasi mengenai penelitian ini)
- Surat persetujuan (untuk ditandatangani jika ibu bersedia untuk berpartisipasi)

***Ibu akan mendapatkan salinan lembar persetujuan ini secara lengkap***

**BAGIAN I: Lembar Informasi**

**Yth. Ibu/Bapak,**

Saya dokter spesialis anak yang bekerja di Rumah Sakit Budi Kemuliaan. Saat ini, kami, bekerja sama dengan Fakultas Kedokteran Universitas Indonesia, RS Cipto Mangunkusumo, dan *University Medical Center* Utrecht, Belanda, sedang melaksanakan penelitian mengenai pemberian ASI (air susu ibu) dan dampaknya bagi kesehatan anak.

Saya akan memberikan informasi mengenai penelitian ini dan mengundang Ibu untuk ikut serta. Ibu tidak harus membuat keputusan saat ini juga apakah akan ikut serta atau tidak. Sebelum Ibu memutuskan, Ibu dapat membahas penelitian ini dengan siapa pun yang ibu inginkan.

Mungkin terdapat beberapa kalimat yang ibu tidak pahami selama saya memberi penjelasan. Jangan sungkan memotong penjelasan saya untuk mengajukan pertanyaan. Saya akan menyediakan waktu untuk menjawab. Jika ada pertanyaan yang muncul kemudian, ibu dapat bertanya pada saya, dokter, atau staf lain yang terlibat dalam penelitian ini.

**Tujuan Penelitian**

Menyusui atau memberi ASI dianggap membawa banyak kebaikan/manfaat bagi bayi. Selain itu, ASI diduga memiliki pengaruh terhadap kesehatan seorang bayi di masa datang, antara lain terhadap timbulnya kelainan jantung/pembuluh darah, seperti serangan jantung atau stroke, ketika bayi/anak tumbuh menjadi dewasa. Melalui penelitian ini, kami ingin mengetahui apakah program optimalisasi (penyempurnaan) pemberian ASI dapat meningkatkan keberhasilan menyusui dan apakah hal ini memengaruhi kondisi jantung/pembuluh darah serta kesehatan bayi/anak pada umumnya.

**Apa yang akan dilakukan pada penelitian ini?**

Pada penelitian ini, kami akan menerapkan program peningkatan pemberian ASI secara terencana, meliputi: (1) penyuluhan; (2) kunjungan rumah, telepon, atau pengiriman SMS oleh perawat/bidan; dan (3) dukungan menyusui pada ibu bekerja. Kesehatan, pertumbuhan, dan perkembangan anak Ibu akan dipantau hingga usia 5 tahun dengan melakukan pemeriksaan berkala. Seluruh program dan pemeriksaan akan dilakukan pada pada kunjungan kehamilan dan imunisasi rutin, kecuali kunjungan anak pada usia 1 dan 5 tahun.

**Peserta penelitian**

Semua ibu hamil tanpa komplikasi yang tidak bermaksud menyusui penuh (tidak memberi minuman atau makanan lain selain ASI) lebih dari 2 bulan setelah kelahiran akan diundang untuk mengikuti penelitian ini.

**Keikutsertaan dalam penelitian ini bersifat sukarela**

Keikutsertaan ibu dalam penelitian ini sepenuhnya bersifat sukarela. Baik ikut serta maupun tidak, semua pelayanan yang ibu terima di rumah sakit ini tidak berubah dan akan berjalan seperti biasa. Ibu berhak berubah pikiran di kemudian hari untuk membatalkan keikutsertaan ibu di penelitian ini.

**Tata Cara penelitian**

1. **Tata Cara khusus**

Mengingat sejauh ini belum diketahui apakah program optimalisasi (penyempurnaan) menyusui lebih baik dibandingkan perawatan biasa dalam meningkatkan pemberian ASI eksklusif dan kesehatan bayi kelak, kami perlu membandingkan dua hal tersebut. Untuk itu, kami **secara acak**, seperti halnya melempar koin, akan membagi peserta penelitian ke dalam dua kelompok. Salah satu kelompok akan mendapat program tambahan/ekstra terkait pemberian ASI (program optimalisasi/penyempurnaan menyusui) di samping pelayanan rutin yang biasa berjalan; kelompok ini disebut dengan kelompok ‘perlakuan’. Adapun kelompok lainnya akan mendapatkan perawatan rutin seperti biasa (kelompok ‘kontrol’).

Untuk mengukur efek optimalisasi (penyempurnaan) pemberian ASI terhadap kesehatan bayi/anak, kami akan melakukan pemeriksaan kesehatan berkala. Di samping pemeriksaan kesehatan rutin, kami akan menilai kondisi pembuluh darah dan jantung dengan menggunakan alat ultrasonografi yang menampilkan gambar jantung/pembuluh darah seperti halnya pemeriksaan janin saat kehamilan. Selain itu, kami juga akan melakukan pemeriksaan fungsi paru dan mengambil usap tenggorok Ibu serta usap saluran napas di bagian belakang hidung bayi/anak. Usap tenggorok/saluran napas akan diambil dengan alat seperti *cotton buds* bergagang lentur. Semua pemeriksaan yang dilakukan tidak menimbulkan nyeri/menyakitkan.

Para petugas kesehatan akan senantiasa memperhatikan ibu dan peserta lainnya dengan teliti selama penelitian berlangsung. Jika ada hal yang mengkhawatirkan atau tidak nyaman bagi Ibu selama proses penelitian, ibu dapat menghubungi saya atau peneliti lain.

1. **Uraian tata cara penelitian**

Pada usia kehamilan 28-32 minggu, kami akan mengundang Ibu untuk berpastisipasi dalam penelitian ini. Jika Ibu bersedia ikut serta, kami akan memasukkan ibu secara acak ke dalam salah satu kelompok penelitian (kelompok perlakuan atau kontrol). Kemudian, di kelompok manapun ibu berada, ibu diminta melanjutkan pemeriksaan kehamilan, melakukan persalinan, dan kunjungan imunisasi anak ibu (pada usia 1, 2, 4, 6, dan 9 bulan) di RS Budi Kemuliaan. Saat anak ibu berusia 1 tahun dan 5 tahun, kami juga akan mengundang anak ibu untuk melakukan pemeriksaan kesehatan. Saat kunjungan imunisasi, kami akan melakukan beberapa pemeriksaan kesehatan seperti yang telah disebutkan di atas. Untuk kepentingan praktis, kami akan menjadwalkan kunjungan ibu/bayi pada hari-hari tertentu.

Jika Ibu termasuk dalam kelompok ‘perlakuan’, ibu akan menerima program tambahan/ekstra terkait pemberian ASI. Program tersebut terdiri dari penyuluhan sebelum, saat, dan setelah kelahiran ketika ibu melakukan kunjungan/pemeriksaan rutin. Seorang bidan juga akan mengunjungi ibu di rumah dalam 3-7 hari pertama dan mungkin minggu kedua setelah kelahiran. Bidan juga akan menelepon dan mengirimkan SMS secara berkala untuk mendukung dan membantu ibu melakukan proses menyusui. Untuk ibu-ibu bekerja, juga akan dilakukan program khusus untuk mendukung kesinambungan menyusui, berupa persiapan untuk kembali bekerja, dukungan di tempat kerja, dan peminjaman alat pompa ASI jika ibu membutuhkannya.

**Lama penelitian**

Penelitian ini akan berlangsung hingga anak berusia satu tahun. Pada usia 5 tahun, kami akan menghubungi ibu kembali dan mengundang anak ibu untuk diperiksakan kesehatannya. Selama jangka waktu tersebut, kami memohon kerja sama ibu untuk senantiasa berhubungan dengan kami dan melakukan kunjungan kesehatan sesuai jadwal yang sudah disepakati.

**Efek Samping**

Semua tata cara dan pengukuran yang kami lakukan pada penelitian ini tidak menimbulkan efek samping atau risiko yang serius untuk kesehatan Ibu dan anak .

**Ketidaknyamanan**

Kami tidak akan pelakukan pemeriksaan yang invasif (menimbulkan rasa sakit). Akan tetapi, pemeriksaan usap saluran napas mungkin akan menyebabkan keluarnya air mata dan menimbulkan rasa tidak nyaman pada anak ibu selama beberapa detik. Pengambilan usap tenggorok juga mungkin memicu rasa ingin muntah selama beberapa detik.

Selain itu, ibu mungkin harus menyediakan waktu khusus untuk penyuluhan dan pemeriksaan kesehatan anak ibu. Ibu juga mungkin membutuhkan usaha lebih untuk datang dan melakukan kunjungan rutin ke Rumah Sakit Budi Kemuliaan pada jadwal yang tertentu. Ibu juga akan kami mohon kesediannya untuk mengisi catatan bulanan mengenai pola menyusui/pemberian makan dan gejala penyakit yang dialami anak.

**Manfaat**

Melalui keikutsertaan dalam penelitian ini, bayi/anak ibu akan mendapatkan pemeriksaan dan pemantauan kesehatan secara rutin dan teratur. Bayi/anak ibu juga akan mendapatkan imunisasi dasar tanpa dipungut biaya.

Ibu mungkin tidak mendapatkan manfaat langsung penelitian ini, akan tetapi keikutsertaan ibu akan sangat membantu kami untuk menjawab pertanyaan penelitian. Selain itu penelitian ini akan memberi manfaat bagi masyarakat dan generasi yang akan datang.

**Insentif**

Kami akan membiayai imunisasi dasar bayi/anak ibu.

**Kerahasiaan**

Mungkin saja ada orang di lingkungan ibu yang mengetahui keikutsertaan ibu di penelitian ini dan mengajukan beberapa pertanyaan. Kami tidak akan memberitahukan jati diri peserta/subyek penelitian ini kepada orang lain. Informasi yang kami dapatkan dari penelitian akan dijaga kerahasiaannya. Informasi mengenai ibu/anak yang dikumpulkan selama penelitian ini tidak akan ditandai dengan nama, melainkan dengan nomor. Hanya tim peneliti yang akan mengetahui nomor ibu dan menyimpan informasi tersebut secara terkunci. Semua informasi tidak akan disampaikan kepada orang lain kecuali jika dokter yang merawat ibu/anak memerlukannya untuk kepentingan pengobatan.

**Hak untuk menolak dan membatalkan keikutsertaan dalam penelitian**

Ibu tidak harus ikut serta dalam penelitian ini. Jika ibu tidak berkenan untuk ikut serta, penolakan ibu tidak akan memengaruhi pelayanan yang diterima di RS Budi Kemuliaan. Ibu dapat membatalkan keikutsertaan dalam penelitian ini kapan pun ibu inginkan tanpa kehilangan hak-hak sebagai pasien. Perawatan kesehatan ibu/anak di RS ini tidak akan terpengaruh dalam bentuk apapun. Keikutsertaan dalam penelitian ini adalah pilihan ibu dan semua hak ibu/anak akan tetap kami hormati.

**Siapa yang dapat ibu hubungi**

Jika ibu memiliki pertanyaan, ibu dapat menyampaikannya saat ini, nanti, atau bahkan setelah penelitian dimulai. Jika ibu ingin bertanya, ibu dapat menghubungi salah satu dari kami:

**Dr. Ria Subekti**, RS Budi Kemuliaan, Jl Budi Kemuliaan No. 1, Jakarta, HP. 085691207240

**Dr. Irma Sapriani, SpA**, RS Budi Kemuliaan, Jl Budi Kemuliaan No. 1, Jakarta, HP. 081389200350

**Dr. Nikmah Salamia Idris, SpA**, RS Budi Kemuliaan/RS Cipto Mangunkusumo, Jakarta, HP. 081310004373

**Proposal ini telah dikaji dan diterima oleh Komite Etik Fakultas Kedokteran Universitas Indonesia, yaitu komite yang bertugas untuk memastikan peserta penelitian terlindung dari bahaya. Jika ibu ingin mengetahui lebih banyak tentang komite etik, silakan menghubungi Komite Etik Fakultas Kedokteran Universitas Indonesia, Jl. Salemba No. 6, Jakarta**

**BAGIAN II: Lembar persetujuan**

**Saya telah terlebih dahulu membaca atau dibacakan informasi mengenai penelitian ini. Saya telah mendapat kesempatan untuk bertanya mengenai penelitian ini dan semua pertanyaan yang saya ajukan telah dijawab dengan memuaskan. Saya berkenan untuk ikut serta sebagai peserta dalam penelitian ini secara sukarela dan mengerti mengenai hak yang saya miliki untuk menarik keikutsertaan saya dari penelitian ini kapan saja saya inginkan tanpa memengaruhi perawatan kesehatan saya.**

Nama Subyek ___________________

Tanda tangan Subyek ___________________

Tanggal ___________________

(tanggal/bulan/tahun)

**Bagi saksi untuk subyek tuna aksara:**

**Saya telah menyaksikan pembacaan lembar informasi kepada calon subyek dengan seksama, dan individu yang bersangkutan telah mendapatkan kesempatan untuk mengajukan pertanyaan. Saya memastikan bahwa yang bersangkutan telah menyatakan kesediannya tanpa paksaan.**

Nama saksi ____________________ DAN Cap Jempol Peserta

Tandatangan saksi ____________________

Tanggal ____________________

(tanggal/bulan/tahun)

**Saya telah membacakan atau menyaksikan pembacaan lembar informasi dengan seksama kepada calon peserta, dan individu yang bersangkutan telah mendapatkan kesempatan untuk mengajukan pertanyaan. Saya memastikan bahwa yang bersangkutan telah memberikan kesediannya tanpa paksaan.**

Nama peneliti _____________________

Tandatangan peneliti _____________________

Tanggal ___________________

(tanggal/bulan/tahun)

**Sebuah salinan dari lembar informasi dan persetujuan ini telah disediakan untuk peserta _____ (diberikan inisial oleh peneliti/asisten peneliti)**
